# Maladie de Leo-Buerger faisant suite à une intoxication au cannabis

**DOI:** 10.11604/pamj.2013.16.82.3450

**Published:** 2013-11-06

**Authors:** Amal Taghy, Badreddine Hassam

**Affiliations:** 1 CHU Ibn Sina, Service de Dermatologie-Vénérologie, Rabat, Maroc; 2 Faculté de Médecine et de Pharmacie, Med V Souissi, Rabat, Maroc

**Keywords:** Léo-Buerger, intoxication, cannabis, vasoconstriction, Léo-Buerger disease, intoxication, cannabis, vasoconstriction

## Image en medicine

La maladie de Léo-Buerger est une artérite du sujet jeune généralement de sexe masculin et classiquement associée au tabac. Sa pathogénie exacte demeure inconnue mais parait-il que le tabac n'est pas le seul facteur toujours incriminé dans sa genèse. Le cannabis est une drogue cultivée en zone équatoriale dont l'association avec la maladie de Léo-Buerger a été décrite la première fois par Sterne en 1960. On en connaît plusieurs sous-types, dont le chanvre indien qui en plus de son effet stupéfiant, sécrète une substance riche en cannabinoïdes dont le D9 tétrahydrocannabitol qui détermine une vasoconstriction périphérique par effet tyramine-like sur les terminaisons nerveuses adrénergiques. La maladie de Léo-Buerger associée au cannabis se caractériserait par un début plus précoce (28 ans), une présentation clinique à type de thromboangéite oblitérante, des lésions très distales, une atteinte fréquente des membres supérieurs et une intoxication tabagique moindre. Il n'existe actuellement pas de traitement spécifique, cependant, le sevrage et les soins locaux sont fondamentaux. L'iloprost intra-veineux peut aider à passer un cap difficile et apporter un bénéfice en termes de diminution des taux d'amputation, mais les problèmes de tolérance et de coût limitent son utilisation. Le recours à d'autres traitements comme les vasodilatateurs classiques, les anti-agrégants plaquettaires, les anti-coagulants, la thrombolyse in situ, les solutés cristalloïdes et colloïdes et la revascularisation chirurgicale ont été utilisés sans grand succès et l'amputation reste indispensable lorsque l'ischémique est irréversible. Nous rapportons le cas d'un jeune homme de 31 ans, non tabagique et consommant en moyenne six joints par semaine de cannabis. L'examen clinique objectivait des claudications intermittentes, une ischémie aiguë distale des membres inférieurs avec abolition des pouls distaux, nécroses et gangrènes plus marquées au niveau du gros orteil droit et du troisième orteil gauche. Le reste de l'examen somatique était normal et il n'y avait pas de phénomène de Raynaud, ni d'ischémie distale des membres supérieurs, ni de symptomatologie articulaire associée. Un bilan à la recherche d'autres facteurs de risques cardio-vasculaires, le dosage des facteurs de coagulation et les examens immunologiques à la recherche d'une vascularite étaient normaux. L'imagerie vasculaire montrait une occlusion des artères pédieuse et tibiale postérieure avec présence de collatérales plantaires en «tire-bouchon». Le traitement consistait à des cures courtes d'Ilosprost cinq jours de suite en milieu hospitalier à une dose de 1,5ng/kg/min et des soins locaux associant des nettoyages, une excision des tissus superficiels nécrosés et des pansements gras, permettant une cicatrisation en quatre mois. Par ailleurs, le sevrage était obtenu graduellement dans un centre spécialisé.

**Figure 1:**
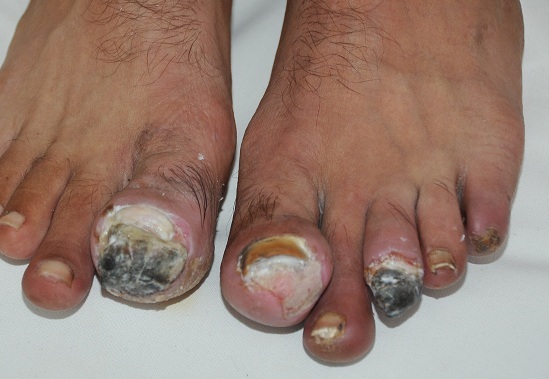
Troubles trophiques à type de gangrène nécrosante plus marquée au niveau du gros orteil droit et du troisième orteil gauche et début d'amputation du cinquième orteil gauche

